# Supplementary material for: Analysing Researchers’ Engagement in Research Hospitals: A Pilot Study in IRCCS—Italian Research Hospitals
Source: Healthcare (Basel). 2022 Dec 5;10(12):2458. doi: 10.3390/healthcare10122458 (PMC9778415; doi:10.3390/healthcare10122458)
Supplement: Supplementary file 1 [file healthcare-10-02458-s001.zip › Supplementary File S1.pdf]

Supplementary File S1: Checklist for Reporting Results of Internet E-Survey (CHERRIES)

**Item Category**	**Checklist Item**	**Page no.**
Design	Describe survey design	3
IRB approval and Informed consent process	IRB approval	NA
	Informed consent	NA
	Data protection	NA
Development and pre-testing	Development and testing	4
Recruitment process and description of the sample having access to the questionnaire	Open survey versus closed surveys	4
	Contact mode	4
	Advertising the survey	4
Survey administration	Web/e-mail	4
	Context	2
	Mandatory/voluntary	4
	Incentive	NA
	Time/Date	4
	Randomization of items or questionnaires	NA
	Adaptive questioning	NA
	Number of items	4
	Number of screens	NA
	Completeness check	NA
	Review step	NA
Response rates	Unique site visitor	6
	View rate (Ratio unique site visitor/unique survey visitors)	NA

	Participation rate (Ratio unique survey page visitors/agreed to participate)	6
	Completion rate (Ratio agreed to participate/finished survey)	6
Preventing multiple entries from the same individual	Cookies	NA
	IP check	NA
	Log file analysis	NA
	Registration	NA
Analysis	Handling of incomplete questionnaires	6
	Questionnaires submitted with an atypical timestamp	NA
	Statistical correction	5
