# Supplementary material for: Analysing Researchers’ Engagement in Research Hospitals: A Pilot Study in IRCCS—Italian Research Hospitals
Source: Healthcare (Basel). 2022 Dec 5;10(12):2458. doi: 10.3390/healthcare10122458 (PMC9778415; doi:10.3390/healthcare10122458)
Supplement: Supplementary file 1 [file healthcare-10-02458-s001.zip › Supplementary File S2.pdf]

Supplementary File S2. *A priori* coding

**Have you ever applied for funding?**	• No<br>• Yes, actively<br>• Yes, indirectly<br>• Both<br><br>Note: answers Yes, actively; Yes, indirectly; Both were coded as ENGAGED
**During the preparation of a project, on which aspect would you prefer to be mostly supported?**	*(Possibility of multiple choice without a limit of answers)*<br>• ***Scouting advice and call selection:*** *ex. tools for tenders research, call congruence evaluation with the proposed research project etc.*<br>• ***Technical-scientific advice:*** *ex. winning design of your project, setting of dissemination of results, enhancement of research through technology transfer, biostatistic support, etc.*<br>• ***Information advice:*** *ex. budget setting etc.*<br><br>Note: at least two answers were coded as ENGAGED
**Would you find reading abstracts of funded projects, together with reviewers' comments as a comparison method?**	• No<br>• Yes<br><br>Note: Yes was coded as ENGAGED

**In addition to your unit head, is there a figure inside the structure with the role of project reviewer in the pre-submission phase?**	• No<br>• Yes<br><br>Note: Yes was coded as ENGAGED
**During project preparation, have you ever analysed the potential long-term impact of the projects through technology transfer activities?**	• No<br>• Yes<br>• Yes, but through the support of an office<br><br>Note: Yes and Yes, but through the support of an office, were coded as ENGAGED
**During project preparation, who are your Institutional contact persons for the assessment of the project potential impact through technology transfer activities?**	• Nobody<br>• Grant Officer<br>• Technology Transfer Officer<br>• Other, specify (free text max 100 characters)<br><br>Note: Nobody was coded as unengaged
**Have you ever participated in internal/external courses/meetings related to the preparation of the project proposals?**	• No<br>• Yes

	Note: Yes was coded as ENGAGED
**Would you be interested in participating in internal/external courses /meetings to support researchers, through online platforms or face-to-face courses?**	• Online platform<br>• Course in presence<br>• Both<br><br>Note: Both was coded as ENGAGED
**Would you have the opportunity to organize face-to-face courses at your facility?**	• No<br>• Yes<br><br>Note: Yes was coded as ENGAGED
**During the preparation of a funding request, would you need planning communication and dissemination activities support?**	• No<br>• Yes<br><br>Note: Yes was coded as ENGAGED
